# CCN5 knockout mice exhibit lipotoxic cardiomyopathy with mild obesity and diabetes

**DOI:** 10.1371/journal.pone.0207228

**Published:** 2018-11-28

**Authors:** Jihwa Kim, Sanghyun Joo, Gwang Hyeon Eom, Seung Hoon Lee, Min-Ah Lee, Miyoung Lee, Ki Woo Kim, Do Han Kim, Hyun Kook, Tae Hwan Kwak, Woo Jin Park

**Affiliations:** 1 College of Life Sciences, Gwangju Institute of Science and Technology, Buk-gu, Gwangju, Republic of Korea; 2 Department of Pharmacology and Medical Research Center for Gene Regulation, Chonnam National University Medical School, Hwasun-gun, Jeollanam-do, Republic of Korea; 3 NadianBio, 201-1 Wonkwang Start-up Assistance Center, Wonkwang University, Iksan, Jeollabuk-do, Republic of Korea; 4 Department of Oral Biology, Yonsei University College of Dentistry, Seodaemun-gu, Seoul, Republic of Korea; Vall d’Hebron Institut de Recerca, SPAIN

## Abstract

Obesity is associated with various human disorders, such as type 2 diabetes, cardiovascular diseases, hypertension, and cancers. In this study, we observed that knockout (KO) of CCN5, which encodes a matricellular protein, caused mild obesity in mice. The CCN5 KO mice also exhibited mild diabetes characterized by high fasting glucose levels and impaired insulin and glucose tolerances. Cardiac hypertrophy, ectopic lipid accumulation, and impaired lipid metabolism in hearts were observed in the CCN5 KO mice, as determined using histology, quantitative RT-PCR, and western blotting. Fibrosis was significantly greater in hearts from the CCN5 KO mice both in interstitial and perivascular regions, which was accompanied by higher expression of pro-fibrotic and pro-inflammatory genes. Both systolic and diastolic functions were significantly impaired in hearts from the CCN5 KO mice, as assessed using echocardiography. Taken together, these results indicate that CCN5 KO leads to lipotoxic cardiomyopathy with mild obesity and diabetes in mice.

## Introduction

The prevalence of obesity has reached epidemic proportions worldwide. Obesity is often accompanied by comorbidities, such as type 2 diabetes, cardiovascular diseases, hypertension, and cancers [1]. In addition to excessive lipid storage in adipose tissue, obesity induces ectopic lipid accumulation in non-adipose tissues, including heart, skeletal muscle, and liver, which leads to insulin resistance and associated metabolic complications in these tissues [2]. In particular, ectopic lipid accumulation in hearts results in cardiac dysfunction and heart failure [3, 4]. The excessive lipid in the circulation also induces greater cardiac lipid uptake and oxidation [5], which, in turn, causes inflammation and oxidative stress eventually leading to cardiac fibrosis and dysfunction [6].

CCN5, also known as WNT1-inducible signaling pathway protein 2 (WISP2), is a member of the CCN family of proteins (members CCN1–6) that encode matricellular proteins with diverse cellular functions. We previously showed that CCN5 inhibits cardiac fibrosis through inhibition of endothelial-to-mesenchymal transition (EndMT) and the trans-differentiation of fibroblasts to myofibroblasts in hearts. The anti-fibrotic effects of CCN5 are thought to be mediated by downregulation of the transforming growth factor β (TGF-β) signaling pathway [7]. CCN5 can also reverse prior fibrotic changes in hearts by inducing apoptosis in myofibroblasts, but not in cardiomyocytes and fibroblasts [7, 8]. We therefore generated whole body CCN5 knockout (KO) mice to further delineate the function of CCN5. The homozygous CCN5 KO mice were viable and fertile, but were obese compared with wild-type (WT) littermates. This finding was not entirely unexpected, given that previous reports demonstrated a role for CCN5 in the regulation of adipogenesis. In adipose tissue, CCN5 was shown to inhibit the developmental commitment of mesenchymal stem cells to form preadipocytes and the differentiation of preadipocytes into mature adipocytes [9, 10].

In this report, we provide compelling lines of evidence showing that CCN5 KO in mice results in mild obesity and diabetes, accompanied by ectopic lipid accumulation in hearts. These observations, combined with the greater cardiac fibrosis and impaired cardiac function, suggest that lipotoxic cardiomyopathy is generated by CCN5 KO.

## Methods

### Animals

The mice were maintained under controlled conditions, and all animal experiments were performed with the approval of the Animal Care Committee of the Gwangju Institute of Science and Technology (approval number: GIST-2016-005). All experiments in this study were performed in accordance with relevant guidelines and regulations. To generate CCN5 KO mice, sperm with a Wisp2^tm1(KOMP)Vlcg^ gene deletion allele was obtained from the trans-NIH (Knock-Out Mouse Project) KOMP Repository (project ID: VG12404). The purchased sperm was used to fertilize ova to obtain offspring on a C57/BL/6 background. For this work, a customized KO mouse generation service (Macrogen, Korea) was used. Mice were fed with normal chow diet (NCD) or high fat diet (HFD) (60% kcal as fat, Research Diets, Inc.; D12492) for 24 weeks starting from 4 weeks of age. Body weights and blood glucose levels were measured every 4 weeks, and food and water intake were measured daily for 5 days at week 22. After completing 24 weeks of feeding with NCD or HFD, mice were sacrificed by cervical dislocation. Blood samples were collected directly from the heart, and organs and tissues were dissected and weighed. HbA1c percentage was determined using an Easy A1c kit and a HbA1c assay device (Infopia Co., Ltd., Osang Healthcare, Korea).

### Insulin tolerance test (ITT) and glucose tolerance test (GTT)

For ITT, mice were fasted for 5 h in the morning and administered with human insulin (Humalog, 0.75 U/kg) by intraperitoneal injection. Blood glucose levels were measured at 0, 15, 30, 60, 90, and 120 min after the injection. For GTT, mice were fasted for 16 h (nocturnal) and intraperitoneally administered with 20% glucose (2 g glucose/kg). Blood glucose levels were then measured at 0, 15, 30, 60, 90, and 120 min after the injection.

### Plasma assays

To measure plasma levels of adiponectin and insulin, blood samples were collected directly from the heart and transferred to EDTA-coated tubes. The plasma fraction of whole blood was obtained using a refrigerated centrifuge (2,500 × *g* for 30 min). Assay kits (Mouse Adiponectin ELISA Kit, KMP0041, Thermo Fisher Scientific; Ultra Sensitive Mouse Insulin ELISA Kit, 90080, Crystal Chem; Total Cholesterol Assay Kit, K603, BioVision; Free Fatty Acid (FFA) Assay Kit, K612, BioVision, Triglyceride (TG) Assay Kit, ab65336, Abcam) were used according to the manufacturer’s instructions.

### Histological analysis

Hearts were extracted, immediately rinsed in cold PBS, and fixed with 10% formalin. The fixed hearts were then embedded in paraffin blocks, and sectioned into 6 μm thick slices. To determine cardiomyocyte cell size, heart sections were stained with hematoxylin (HHS32, Sigma) and eosin Y solution (HT110116, Sigma). To estimate the size of the fibrotic areas, heart sections were stained with a Masson’s Trichrome Kit (HT15, Sigma). For oil red O staining, frozen heart sections were prepared and stained with oil red O working solution diluted from 0.5% oil red O solution (Sigma; O1516) for 1 h. Subsequently, the sections were counterstained with hematoxylin solution (Sigma; HHS32) for 10 sec and scanned at 40× magnification using an Aperio ScanScope Slide Scanner (Aperio Technologies, Inc.). Five regions on each section were chosen, and the captured images were used for quantification using ImageJ software.

### Quantitative real-time (qRT)-PCR

Total RNA from heart tissues was isolated using TRI Reagent (MRC; TR118). Total RNA from adipose tissues was isolated using RNeasy Mini Kit (74104, QIAGEN). Total cDNA was obtained by reverse transcription using Improm-2 reverse transcriptase (Promega; A3802) with oligo-dT primers. Real-time PCR was performed using the TaKaRa Real Time System, according to the manufacturer’s instructions, and SYBR Premix Ex Taq (Tli RNaseH Plus; TaKaRa; RR420A). The primer sequences are shown in S1 Table.

### Western blot analysis

Cell lysates (50 μg) were separated on SDS-PAGE gels and transferred to PVDF membranes (Millipore). The membranes were blocked with 5% skim milk solution, and incubated with primary antibodies at 4°C overnight. Antibodies targeting phospho-Akt (Ser473, 9271S; Thr308, 9275S), total Akt (9272S), phospho-GSK3β (Ser9, 9336S), total GSK3β (9315S), and GAPDH (2118S) were purchased from Cell Signaling Technologies, Inc. The membranes were then incubated with horseradish peroxidase-conjugated secondary antibodies (Invitrogen) and visualized using the Western ECL solution kit (FEMTO, LPS solution).

### Echocardiographic assessment

To determine cardiac function, mice were anesthetized with avertin (250 mg/kg). Echocardiography was performed using the GE Vivid S5 Cardiovascular Ultrasound System (GE Healthcare) with a 15 MHz microprobe. M-mode measurements of the left ventricle were performed using the long-axis view of the 2-dimensional mode. Amplitudes of E and A of the mitral valve flow wave were also measured, and the velocity of the E and A waves and their ratio were automatically calculated.

### Statistics

Data are shown as mean ± SD. The statistical significance of any differences between groups was assessed using Student’s *t*-test or one-way ANOVA followed by Bonferroni’s correction for comparison of means. *P*<0.05 was considered to represent statistical significance.

## Results

### CCN5 KO mice exhibit mild obesity

**Fig 1:**
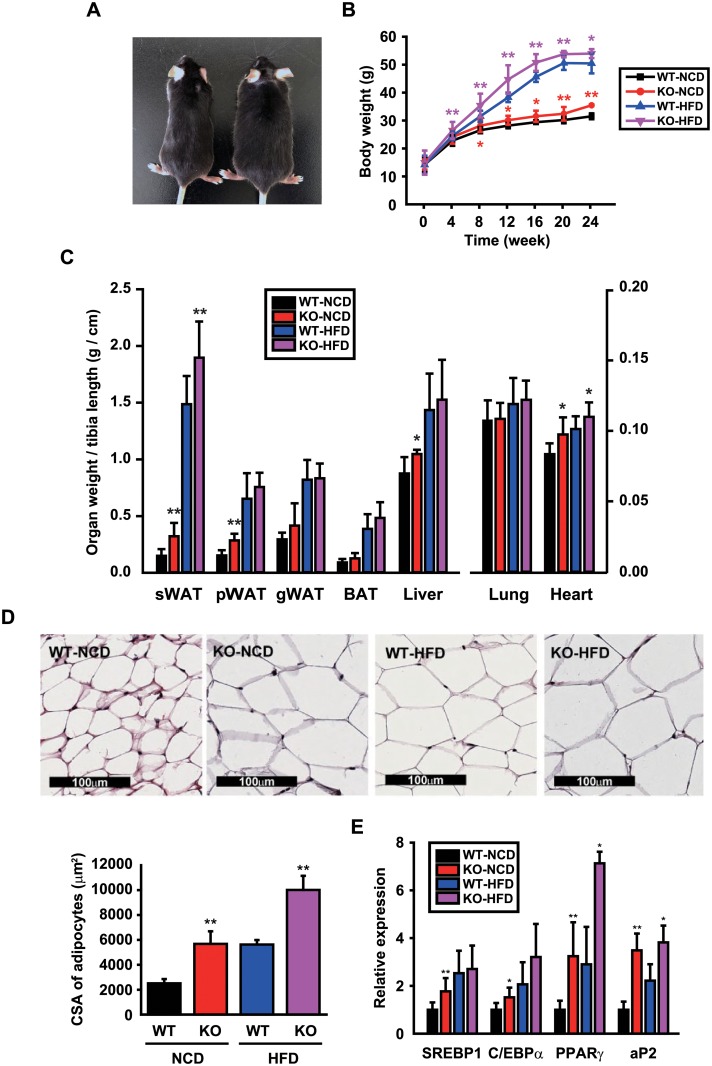
CCN5 KO mice exhibit mild obesity. (A) A representative image of 24-week-old WT and CCN5 KO mice. (B) Body mass changes in each group during the 24 weeks of NCD or HFD feeding (mean data collected from 3 independent experimental sets, n = 6–12 per group for each experimental set). (C) Organ masses of WT and CCN5 KO mice after 24 weeks of NCD or HFD feeding (n = 7 for WT-NCD group, n = 6 for KO-NCD group, n = 14 for WT-HFD group, n = 16 for KO-HFD group). sWAT, subcutaneous white adipose tissue; pWAT, perirenal white adipose tissue; gWAT, gonadal white adipose tissue; iBAT, interscapular brown adipose tissue. (D) Representative H&E staining images of sWAT sections obtained from WT and CCN5 KO mice (upper panel). Mean cross-sectional areas of adipocytes (n = 3 per group) (lower panel). (E) The mRNA expression levels of adipogenic genes, sterol regulatory element-binding protein 1 (SREBP1), CCAAT/enhancer-binding protein α (C/EBPα), peroxisome proliferator activated receptor γ (PPARγ), and adipocyte protein 2 (aP2) (n = 4–6 per group). Data are presented as mean ± SD. *p<0.05, **p<0.01 *vs*. WT.

The CCN5 KO mice used in this study contain the Wisp2^tm1(KOMP)Vlcg^ deletion allele that was generated by the trans-NIH KOMP. This allele was thought to be null due to deletion of exons 2–5, and this was confirmed by qRT-PCR analysis (S1 Fig). The appearance and behavior of KO mice were grossly normal, but their body size was larger at the age of 5–6 months (Fig 1A). We therefore monitored the body masses of WT and KO mice that were fed with NCD or HFD for 24 weeks. KO mice showed slight but consistent obesity compared with WT mice when fed with NCD, and larger differences in body masses were observed between WT and KO mice when fed with HFD (Fig 1B). There was no discernible difference in daily food intake between WT and KO mice when fed with NCD, but this was slightly higher in KO than WT mice when fed with HFD (S2 Fig). Organs and tissues including white adipose tissue (WAT), brown adipose tissue (BAT), liver, lung, heart, and tibia were removed and weighed at the end of the experiment. The masses of the subcutaneous WAT (sWAT), perirenal WAT (pWAT), liver, and heart, as normalized by the length of tibia, were significantly greater in KO than WT mice (Fig 1C). The obtained sWAT was sectioned and stained with hematoxylin & eosin (H&E). Cross-sectional area (CSA) of adipocytes in sWAT sections was significantly larger in KO than WT mice (Fig 1D). The mRNA expression levels of adipogenic genes including sterol regulatory element-binding protein 1 (SREBP1), CCAAT/enhancer-binding protein α (C/EBPα), peroxisome proliferator activated receptor γ (PPARγ), and adipocyte protein 2 (aP2) were significantly higher in KO than WT mice (Fig 1E). Collectively, these data showed that CCN5 KO leads to mild obesity in mice.

### CCN5 KO mice exhibit mild type 2 diabetes

**Fig 2:**
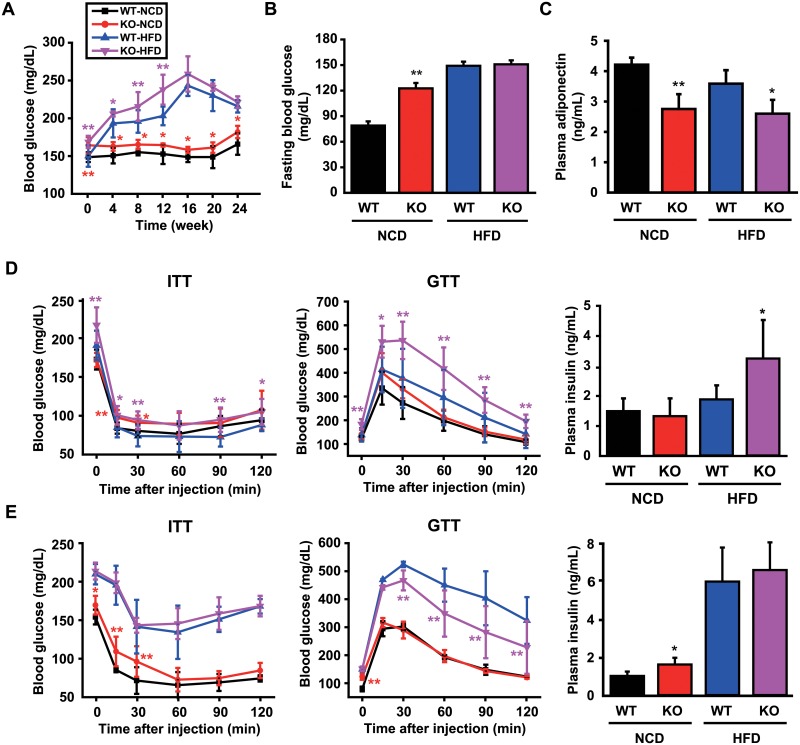
CCN5 KO mice exhibit mild type 2 diabetes. (A) Blood glucose levels during the 24 weeks of NCD or HFD feeding (mean data collected from 3 independent experimental sets, n = 6–12 per group for each experimental set). (B) Fasting blood glucose levels in each group after 16 h of nocturnal fasting (mean data collected from 3 independent experimental sets, n = 6–12 per group for each experimental set). (C) Plasma adiponectin levels in each group (n = 4 for NCD group, n = 6 for HFD group). (D) ITT and GTT after 11 weeks of NCD or HFD feeding (n = 8–9 per group for each group). Plasma insulin levels after 12 weeks of NCD or HFD feeding (n = 7 per group). (E) ITT and GTT after 23 weeks old NCD or HFD feeding (mean data collected from 3 independent experimental sets, n = 6–8 per group for each experimental set). Plasma insulin levels after 24 weeks of NCD or HFD feeding (n = 7–8 per group). Data are presented as mean ± SD. *p<0.05, **p<0.01 *vs*. WT.

We next examined whether the obese phenotypes observed in the CCN5 KO mice are associated with diabetes. Blood glucose levels were significantly higher in KO than WT mice throughout the period of NCD feeding. KO mice fed with HFD exhibited significantly higher glucose levels until week 12 (Fig 2A). Fasting blood glucose levels, measured after 16 h of nocturnal fasting, were determined after mice had been fed with NCD or HFD for 24 weeks. These were significantly higher in KO than WT mice fed with NCD, but not when fed with HFD (Fig 2B). Plasma adiponectin levels were significantly lower in KO than WT mice as determined by ELISA (Fig 2C), which was consistent with the obese phenotypes observed in KO mice (Fig 1).

ITT and GTT were performed to evaluate the capability for the insulin-stimulated reduction of blood glucose level and the disposal of the injected glucose, respectively, after 12 and 24 weeks of diet feeding. At week 12, ITT (Fig 2D, Left) and GTT (Fig 2D, Middle) were significantly impaired in KO than WT mice fed with HFD. In addition, plasma insulin levels were significantly higher in KO than WT mice fed with HFD (Fig 2D, Right). At week 24, no overall significant differences in ITT and GTT were observed between KO and WT mice (Fig 2E, Left and Middle), but a significant impairment in ITT in KO mice fed with NCD was observed within 30 min after the insulin administration. Plasma insulin level was also higher in KO mice fed with NCD (Fig 2E, Right). These data suggest that CCN5 KO leads to mild type 2 diabetes in mice.

### CCN5 KO mice exhibit lipotoxic cardiomyopathy

**Fig 3:**
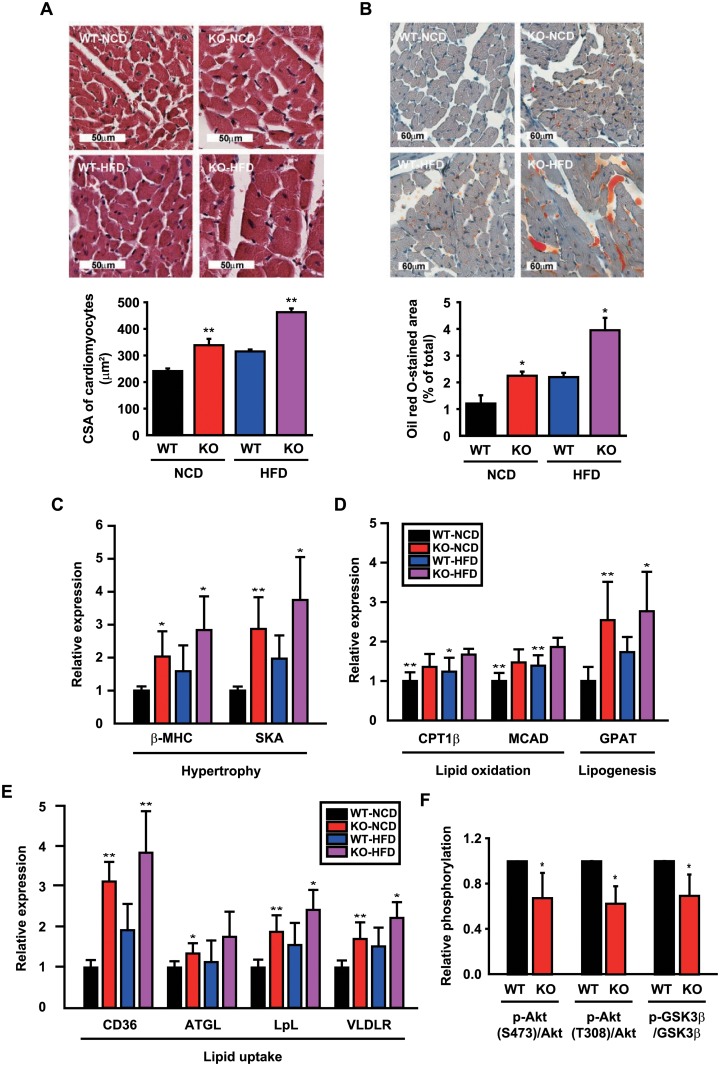
CCN5 KO exhibits lipotoxic cardiomyopathy. (A) Representative images and quantitation of CSA of H&E-stained heart sections from WT and CCN5 KO mice after 24 weeks of NCD or HFD feeding (n = 4–7 per group). (B) Representative images and quantitation of the stained area of oil red O-stained heart sections from WT and CCN5 KO mice after 24 weeks of NCD or HFD feeding (n = 4 per group). (C-E) Relative mRNA expression levels of cardiac hypertrophy- and lipid metabolism-associated genes (n = 4–6 per group). (F) Relative phosphorylation levels of Akt and GSK3β in isolated cardiomyocytes treated with insulin (n = 4 per group). Original blot images are shown in S3 Fig. Data are presented as mean ± SD. *p<0.05, **p<0.01 *vs*. WT.

Hearts obtained from the experiments shown in Fig 1C were further subjected to histological and molecular biological analyses. H&E staining of heart sections and measurements of CSA revealed that CCN5 KO led to cardiac hypertrophy (Fig 3A). Oil red O staining of the heart sections also revealed ectopic accumulation of lipids in hearts from KO mice (Fig 3B). Therefore, the greater heart mass in KO mice (Fig 1C) is likely to be contributed to by both the hypertrophy of cardiomyocytes and the accumulation of lipids in hearts. The mRNA expression levels of the cardiac hypertrophy-associated genes including β-myosin heavy chain (β-MHC) and skeletal actin (SKA) were significantly higher in hearts from KO than WT mice (Fig 3C). The mRNA expression levels of lipid oxidation-associated genes including carnitine palmitoyl transferase 1β (CPT1β), medium chain acyl-CoA dehydrogenase (MCAD) and a triglyceride synthesis-associated gene, glycerol-3-phosphate acyltransferase (GPAT), were significantly higher in hearts from KO mice (Fig 3D). In addition, the fatty acid uptake-associated genes including CD36, adipose triglyceride lipase (ATGL), lipoprotein lipase (LpL), and very-low-density lipoprotein receptor (VLDLR) were significantly higher in hearts from KO than WT mice (Fig 3E). Cholesterol level was significantly higher in plasma from KO mice fed with HFD, and triglyceride level was significantly higher in cardiomyocytes from KO mice fed with NCD (S2 Table).

Treatment of isolated cardiomyocytes with insulin activates a down-stream insulin signaling pathway, which can be monitored by the increased phosphorylation of AKT serine/threonine kinase 1 (AKT1) at Ser 473 and Thr 308 and glycogen synthase kinase 3β (GSK-3β) at Ser 9. This insulin-induced hyper-phosphorylation of AKT1 and GSK-3β was significantly reduced in KO mice (Fig 3F) as assessed using western blotting (S3 Fig), which implied that mild insulin resistance was induced in KO mice. The heart condition defined as lipotoxic cardiomyopathy is characterized by cardiac hypertrophy, ectopic lipid accumulation, and insulin resistance. Therefore, our data suggest that lipotoxic cardiomyopathy is generated as a result of CCN5 KO in mice.

### CCN5 KO leads to cardiac fibrosis

**Fig 4:**
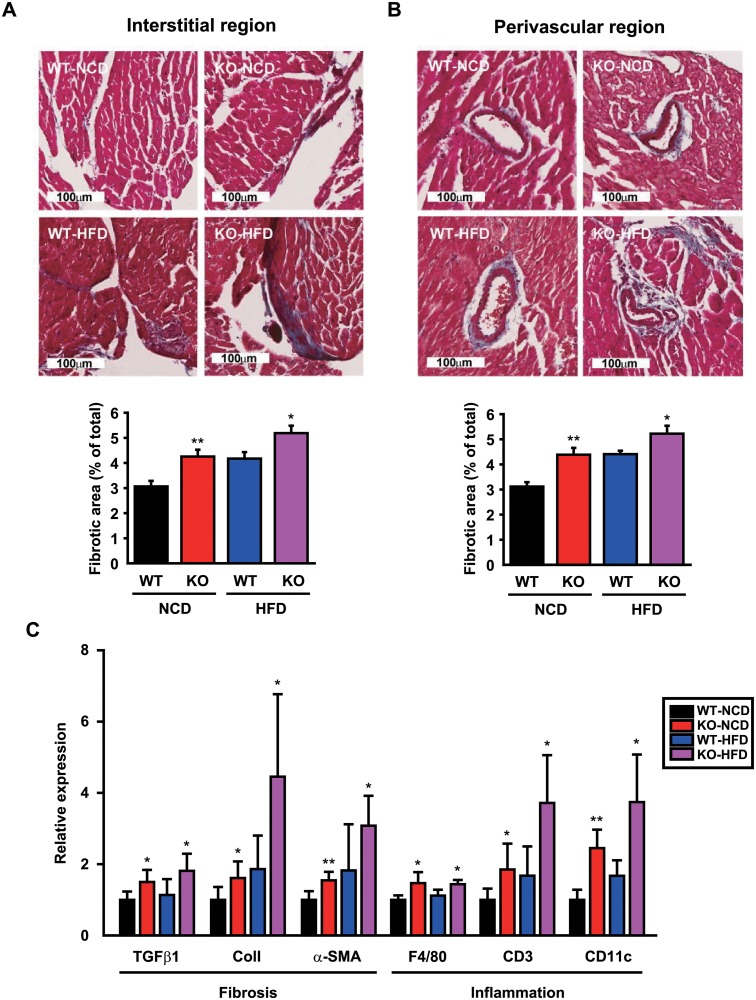
CCN5 KO leads to cardiac fibrosis. (A and B) Representative images and measurements of fibrotic areas for Masson’s Trichrome-stained heart sections from WT and KO mice after 24 weeks of NCD or HFD feeding (n = 4–7 per group). (C) Relative mRNA expression levels of fibrosis- and inflammation-associated genes (n = 6–7 per group and n = 6 per group, respectively). Data are presented as mean ± SD. *p<0.05, **p<0.01 *vs*. WT.

Excessive lipid accumulation is also associated with cardiac fibrosis in lipotoxic cardiomyopathy. Trichrome staining of heart sections revealed significant collagen depositions in interstitial (Fig 4A) and perivascular (Fig 4B) areas in KO than WT mice. The mRNA expression levels of the fibrosis-related genes including TGF-β1, collagen 1 (Col1), and α-smooth muscle actin (α-SMA) and the inflammation-related genes including F4/80, CD3, and CD11c were higher in KO than WT mice (Fig 4C). These results indicate that CCN5 KO leads to cardiac fibrosis. Intriguingly, trichrome staining of sWAT sections also revealed prominent fibrosis in both interstitial and perivascular areas of adipocytes in KO than WT mice (S4 Fig).

### CCN5 KO leads to cardiac dysfunction

**Fig 5:**
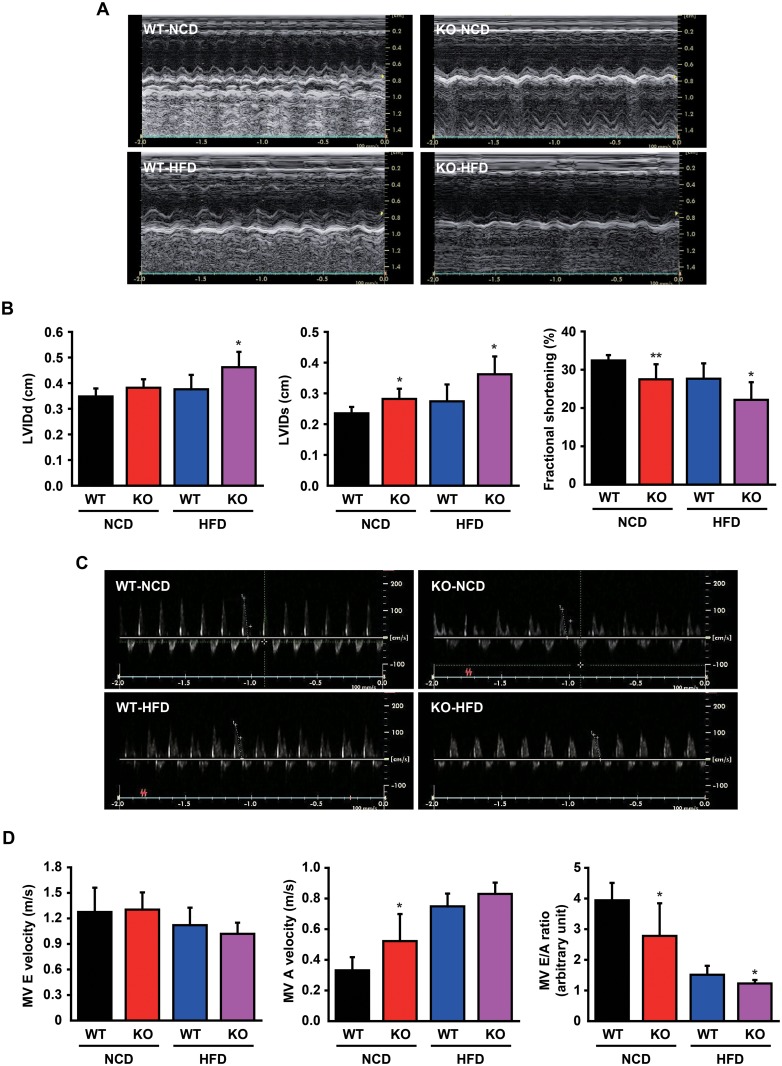
CCN5 KO leads to cardiac dysfunction. (A) Representative M-mode echocardiography images of WT and KO mice after 24 weeks of NCD or HFD feeding. (B) Parameters for cardiac remodeling and function. LVIDd, left ventricular internal dimension at diastole; LVIDs, left ventricular internal dimension at systole (n = 7 for NCD group, n = 9 for HFD group). (C) Representative Doppler echocardiography images of WT and KO mice after 24 weeks of NCD or HFD feeding. (D) Parameters for diastolic function. Mitral valve (MV) velocities of E and A waves and the E/A ratio (n = 7 for NCD group, n = 8 for HFD group). Data are presented as mean ± SD. *p<0.05, **p<0.01 *vs*. WT.

Abnormal regulation of lipid metabolism and fibrosis are highly associated with cardiac dysfunction. We therefore evaluated cardiac function of KO and WT mice after 24 weeks of diet feeding using M-mode echocardiography (Fig 5A). KO mice exhibited systolic dysfunction with left ventricular dilatation and reduced fractional shortening (Fig 5B; S3A Table). In addition, mitral valve (MV) E and A wave velocities were measured (Fig 5C). The reduced E/A ratio in KO mice implied that CCN5 KO led to diastolic dysfunction (Fig 5D; S3B Table). Collectively, these data indicate that CCN5 KO mice exhibit arrays of lipotoxic cardiomyopathy phenotypes including cardiac hypertrophy, ectopic lipid accumulation, fibrosis, and cardiac dysfunction.

## Discussion

Abnormal metabolism in hearts, which occur in response to various insults including obesity and diabetes, is highly associated with heart failure [11, 12]. Excess lipids are normally stored in adipocytes. However, when the storage capacity of adipocytes is reached, lipids begin to accumulate in non-adipose tissues including the heart [13, 14]. The ectopic lipid accumulation in hearts can be cytotoxic and cause cardiac dysfunction, which can lead to lipotoxic cardiomyopathy [15, 16].

In our previous studies, we demonstrated that CCN5 inhibits cardiac fibrosis by inhibiting EndMT and fibroblast-to-myofibroblast trans-differentiation, two critical processes involved in cardiac fibrosis. In addition, CCN5 induces apoptosis specifically in myofibroblasts, but not cardiomyocytes or fibroblasts. CCN5 activates the intrinsic apoptotic pathway in myofibroblasts by inhibiting the activity of an anti-apoptotic molecule, NFκB [8]. These anti-fibrotic activities of CCN5 were thought to also be at least partially contributed to by its ability to inhibit the TGF-β signaling pathway.

It is noteworthy that TGF-β signaling is implicated in the progression of obesity and diabetes. The expression and plasma levels of TGF-β were found to be high in the adipose tissue of obese mice and diabetic patients, respectively [17, 18]. TGF-β1 was also shown to regulate expression of the insulin gene and pancreatic function [19]. Smad3 is a downstream signaling molecule that mediates TGF-β signaling, and deficiency of Smad3 protects mice from HFD-induced obesity and diabetes through regulation of adipocyte differentiation, and glucose and lipid metabolism [20–23]. Therefore, it is conceivable that CCN5 may exert its anti-obesity effects at least partially by inhibiting the TGF-β signaling pathway.

Direct roles of CCN5 in adipogenesis have well been described. CCN5 has a signal peptide at its amino terminus, thus it is thought to be a secreted protein. However, CCN5 is localized in cytosol and nucleus as well as extracellular space. In cytosol, CCN5 binds to and prevent the nuclear translocation of a transcription cofactor Zfp423 that plays an essential role for the expression of PPARγ. It is well known that adipogenic differentiation is under the control of PPARγ. Thus, trapping of Zfp423 in the cytosol by CCN5 is thought to prevent the expression of PPARγ and consequently to prevent adipogenic commitment of precursor cells (10). In addition, the secreted CCN5 maintains mesenchymal precursor cells in an undifferentiated state through activation of canonical WNT signaling pathway, thus inhibits adipogenic differentiation of the precursor cells (9). These studies suggest that CCN5 may inhibit adipogenesis and obesity in two ways, intracellular and extracellular. The phenotypes of the CCN5 KO mice characterized in this study is largely consistent with the proposed anti-obesity function of CCN5.

Overexpression of CCN5 in the adipose tissue of mice induced hyperplasia of cardiomyocytes, as well as of WAT and BAT, and prevented HFD-induced insulin resistance [24]. This finding was unexpected because overexpression of CCN5 in the heart did not induce hyperplasia or hypertrophy in cardiomyocytes [7]. Instead, transgene- or virus-mediated overexpression inhibited cardiac hypertrophy and cardiac fibrosis [8]. It is possible that cardiac hyperplasia is induced by an unknown mediator that is induced by CCN5 in adipose tissue, which has its effect alone or in combination with CCN5. Identification of such unknown pro-proliferative factors might be of great clinical value because the proliferation of cardiomyocytes can be highly desirable for the treatment of degenerative heart conditions such as myocardial infarction.

Lipotoxic cardiomyopathy is of significant clinical concern. Mouse models of lipotoxic cardiomyopathy have been generated by overexpression of several metabolic genes under the control of cardiac-specific α-MHC promoter. For example, overexpression of LpL or PPARγ in hearts induced lipotoxic cardiomyopathy phenotypes including ectopic cardiac lipid accumulation and dilated cardiomyopathy [25, 26]. There was no reduction in glucose uptake and glycogen storage in these mice. PPARα transgenic mice exhibited arrays of diabetic cardiomyopathy phenotypes including cardiac hypertrophy, systolic dysfunction, and reduced glucose uptake and oxidation [27, 28]. Although we did not direct compare these models in this study, our data indicate that the CCN5 KO mice can also be a valuable model for lipotoxic cardiomyopathy that is distinct from other models with prominent fibrosis and diastolic dysfunction.

It is of note that our KO mice are globally deficient of CCN5. The CCN5 KO mice exhibited prominent fibrosis in sWAT (S4 Fig) as well as hearts (Fig 4). It is possible that CCN5 is involved in fibrosis in other tissues as well. Therefore, tissue-specific KOs would be needed to dissect the roles of CCN5 in specific tissues. For example, it would be interesting to see if CCN5 KO specifically in hearts also leads to lipotoxic cardiomyopathy to a comparable level seen in the whole body CCN5 KO. Nonetheless, our KO mice provide a valuable tool for the study of lipotoxic cardiomyopathy and/or mild obesity and diabetes.

## Supporting information

S1 Fig mRNA expression levels of CCN5. (A) Relative expression of CCN5 in total RNA extracted from organs of WT, CCN5 hetero KO (+/−), and CCN5 KO (−/−) (n = 2 per group). (B) Relative mRNA expression of CCN5 in total RNA extracted from hearts of WT and CCN5 KO mice after completing 24 weeks on a NCD or HFD (n = 4–5 per group). Data are presented as mean ± SD. *p<0.05, **p<0.01 *vs*. WT.
(EPS)

S2 Fig Food intake, water intake, and HbA1c percentage. (A) Mean daily food intake and water intake over 5 days after 22 weeks on NCD or HFD feeding (mean data collected from three experimental sets, n = 6–12 per group for each individual experimental set). (B) HbA1c percentage after 24 weeks on NCD or HFD feeding (n = 4–6/group). Data are presented as mean ± SD. *p<0.05, **p<0.01 *vs*. WT.
(EPS)

S3 Fig Western blot analysis of insulin signaling molecules in isolated cardiomyocytes. Western blot analysis of insulin signaling molecules in isolated adult mouse cardiomyocytes after treatment with human insulin (100 nM) for various periods of time. (B) Original uncropped blot images. Blots marked with protein sizes were used for analysis (blots not marked with protein sizes show irrelevant samples extracted from non-myocytes).
(EPS)

S4 Fig Histological analysis of fibrotic area in adipose tissues. (A) Representative images and (B) quantitative data for Masson’s trichrome staining of adipose tissue sections from WT and CCN5 KO mice. Data are presented as mean ± SD. *p<0.05, **p<0.01 *vs*. WT.
(EPS)

S1 Table Primer sequences for quantitative rear-time PCR. (DOCX)

S2 Table Lipids in plasma and cardiac cells. (DOCX)

S3 Table Doppler’s Echocardiographic data. (DOCX)
