# Supplementary material for: CCN5 knockout mice exhibit lipotoxic cardiomyopathy with mild obesity and diabetes
Source: PLoS One. 2018 Nov 28;13(11):e0207228. doi: 10.1371/journal.pone.0207228 (PMC6261567; doi:10.1371/journal.pone.0207228)
Supplement: S1 Table — (DOCX) [file pone.0207228.s005.docx]

**S1 Table. Primer Sequences for Quantitative Real-Time PCR**

| Gene | Sequence |
| --- | --- |
| 18S rRNA | 5’- GTAACCCGTTGAACCCCATT -3’  5’- CCATCCAATCGGTAGTAGCG -3’ |
| β-MHC | 5′-GACGAGGCAGAGCAGATCGC -3’  5′-GGGCTTCACAGGCATCCTTAGCC-3′ |
| SKA | 5′-TCAGGCGGTGCTGTCTCTCT-3′  5′-TCCCCAGAATCCAACACGAT-3′ |
| CD36 | 5’- TCCTCTGACATTTGCAGGTCTATC -3’  5’- AAAGGCATTGGCTGGAAGAA -3’ |
| CPT1β | 5’- TGCCTTTACATCGTCTCCAA -3’  5’- GGCTCCAGGGTTCAGAAAGT -3’ |
| MCAD | 5’- ACTGACGCCGTTCAGATTTT –3’  5’- GCTTAGTTACACGAGGGTGATG -3’ |
| GPAT | 5’- ATCTTCAGAACAGCAAAATCGAAA  5’- CAGCGGAAAACTCCAAATCC |
| TGF-β1 | 5′- CAACAATTCCTGGCGTTACCTTGG -3′  5′- GAAAGCCCTGTATTCCGTCTCCTT -3′ |
| Collagen 1 | 5′- CCCAAGGAAAAGAAGCACGTC -3′  5′- AGGTCAGCTGGATAGCGACATC -3′ |
| α-SMA | 5′- GCCCAGCCAAGCACTGTCAGGA -3′  5′- TCCCACCATCACCCCCTGATGTC -3′ |
| F4/80 | 5’- CTTGGCTATGGGCTTCCAGTC -3’  5’- GCAAGGAGGACAGAGTTTATCGTG -3’ |
| CD3 | 5’- TGCCTCAGAAGCATGATAAGC -3’  5’- GCCCAGAGTGATACAGATGTCAA -3’ |
| CD11c | 5’- ACACAGTGTGCTCCAGTATGA -3’  5’- GCCCAGGGATATGTTCACAGC -3’ |
| ATGL | 5’- CAACGCCACTCACATCTACGG -3’  5’- GGACACCTCAATAATGTTGGCAC -3’ |
| LpL | 5’- GGACGGTAACGGGAATGTATG -3’  5’- ACGTTGTCTAGGGGGTACTTAAA -3’ |
| VLDLR | 5’- ATGAAGACTGTGCGGATGGC -3’  5’- CGGGGATACACTGAGTAGAACG -3’ |
| SREBP1 | 5’- GGAGCCATGGATTGCACATT -3’  5’- GGCCCGGGAAGTCACTGT -3’ |
| C/EBPα | 5’- CAAAGCCAAGAAGTCGGTGGACAA -3’  5’- TCATTGTGACTGGTCAACTCCAGC -3’ |
| PPARγ | 5’- CCACCAACTTCGGAATCAGCT -3’  5’- TTTGTGGATCCGGCAGTTAAGA -3’ |
| aP2 | 5’- AAGGTGAAGAGCATCATAACCCT -3’  5’- TCACGCCTTTCATAACACATTCC -3’ |
