# Supplementary material for: CCN5 knockout mice exhibit lipotoxic cardiomyopathy with mild obesity and diabetes
Source: PLoS One. 2018 Nov 28;13(11):e0207228. doi: 10.1371/journal.pone.0207228 (PMC6261567; doi:10.1371/journal.pone.0207228)
Supplement: S2 Table — (DOCX) [file pone.0207228.s006.docx]

**S2 Table. Lipids in plasma and cardiac cells**

| **Plasma lipids** | **WT-NCD** | **KO-NCD** | **P-value** | **WT-HFD** | **KO-HFD** | **P-value** |
| --- | --- | --- | --- | --- | --- | --- |
| Cholesterol (mg/dL) | 188.11±12.19 | 222.45±20.37 | 0.13 | 362.88±27.46 | 411.65±30.19* | 0.03 |
| FFA (mmol/L) | 0.33±0.12 | 0.42±0.15 | 0.32 | 0.45±0.15 | 0.65±0.21 | 0.17 |
| TG (mmol/L) | 1.89±0.37 | 2.25±0.65 | 0.30 | 2.08±0.54 | 2.69±0.43 | 0.14 |
| **Intracellular lipid** | **WT-NCD** | **KO-NCD** | **P-value** | **WT-HFD** | **KO-HFD** | **P-value** |
| TG in myocytes  (mg/g protein) | 116.36±19.14 | 184.05±21.48* | 0.02 | 156.72±10.36 | 186.88±20.27 | 0.16 |
| TG in non-myocytes  (mg/g protein) | 18.37±2.39 | 20.53±1.94 | 0.42 | 24.71±10.11 | 27.33±14.76 | 0.86 |
