# Supplementary material for: CCN5 knockout mice exhibit lipotoxic cardiomyopathy with mild obesity and diabetes
Source: PLoS One. 2018 Nov 28;13(11):e0207228. doi: 10.1371/journal.pone.0207228 (PMC6261567; doi:10.1371/journal.pone.0207228)
Supplement: S3 Table — (DOCX) [file pone.0207228.s007.docx]

**S3 Table. Doppler’s Echocardiographic Data**

| **Parameter** | **Unit** | **WT-NCD** | **KO-NCD** | **P-value** | **WT-HFD** | **KO-HFD** | **P-value** |
| --- | --- | --- | --- | --- | --- | --- | --- |
| IVsd | cm | 0.067±0.025 | 0.073±0.010 | 0.584 | 0.084±0.010 | 0.077±0.023 | 0.462 |
| IVSs | cm | 0.114±0.014 | 0.113±0.010 | 0.893 | 0.139±0.016 | 0.116±0.019* | 0.031 |
| LVIDd | cm | 0.348±0.031 | 0.382±0.033 | 0.119 | 0.376±0.056 | 0.462±0.060* | 0.029 |
| LVIDs | cm | 0.235±0.021 | 0.282±0.033* | 0.017 | 0.274±0.055 | 0.362±0.058* | 0.023 |
| LVPWd | cm | 0.089±0.029 | 0.078±0.008 | 0.414 | 0.090±0.025 | 0.069±0.012 | 0.065 |
| LVPWs | cm | 0.116±0.026 | 0.093±0.005 | 0.061 | 0.117±0.021 | 0.101±0.020 | 0.177 |
| EDV | ml | 0.099±0.040 | 0.127±0.045 | 0.261 | 0.143±0.069 | 0.209±0.100 | 0.179 |
| ESV | ml | 0.033±0.013 | 0.052±0.025 | 0.105 | 0.059±0.042 | 0.104±0.055 | 0.107 |
| EF | % | 67.853±2.010 | 60.173±6.493* | 0.012 | 60.363±6.679 | 50.767±8.117* | 0.033 |
| SV | ml | 0.072±0.019 | 0.070±0.021 | 0.895 | 0.084±0.030 | 0.104±0.050 | 0.384 |
| %FS | % | 32.426±1.383 | 27.490±3.933* | 0.010 | 27.647±4.006 | 22.129±4.611* | 0.034 |

**A**

**B**

| **Parameter** | **Unit** | **WT-NCD** | **KO-NCD** | **P-value** | **WT-HFD** | **KO-HFD** | **P-value** |
| --- | --- | --- | --- | --- | --- | --- | --- |
| E Vel | m/s | 1.276±0.285 | 1.304±0.202 | 0.970 | 1.121±0.205 | 1.019±0.131 | 0.271 |
| DecT | ms | 40.077±8.582 | 40.730±9.975 | 0.485 | 48.698±7.715 | 50.513±3.337 | 0.572 |
| Dec Slope | m/s^2^ | 33.927±13.167 | 33.084±7.459 | 0.616 | 24.187±8.820 | 20.193±2.462 | 0.267 |
| A Vel | m/s | 0.331±0.086 | 0.522±0.176* | 0.014 | 0.749±0.083 | 0.830±0.074 | 0.062 |
| E/A Ratio | a.u. | 3.943±0.566 | 2.780±1.066* | 0.024 | 1.511±0.293 | 1.227±0.115* | 0.030 |
